# A Facile Route to Tailoring Peptide-Stabilized Gold Nanoparticles Using Glutathione as a Synthon

**DOI:** 10.3390/molecules19056754

**Published:** 2014-05-23

**Authors:** Rosina Ho Wu, Tan P. Nguyen, Grant W. Marquart, Thomas J. Miesen, Theresa Mau, Marilyn R. Mackiewicz

**Affiliations:** Department of Chemistry, Portland State University, Portland, OR 97201, USA

**Keywords:** peptide-functionalized, glutathione, gold nanoparticles, bioconjugation, ultracentrifugation, surface modification

## Abstract

The preparation of gold nanoparticles (AuNPs) of high purity and stability remains a major challenge for biological applications. This paper reports a simple synthetic strategy to prepare water-soluble peptide-stabilized AuNPs. Reduced glutathione, a natural tripeptide, was used as a synthon for the growth of two peptide chains directly on the AuNP surface. Both nonpolar (tryptophan and methionine) and polar basic (histidine and dansylated arginine) amino acids were conjugated to the GSH-capped AuNPs. Ultracentrifugation concentrators with polyethersulfone (PES) membranes were used to purify precursor materials in each stage of the multi-step synthesis to minimize side reactions. Thin layer chromatography, transmission electron microscopy, UV-Visible, ^1^H-NMR, and fluorescence spectroscopies demonstrated that ultracentrifugation produces high purity AuNPs, with narrow polydispersity, and minimal aggregation. More importantly, it allows for more control over the composition of the final ligand structure. Studies under conditions of varying pH and ionic strength revealed that peptide length, charge, and hydrophobicity influence the stability as well as solubility of the peptide-capped AuNPs. The synthetic and purification strategies used provide a facile route for developing a library of tailored biocompatible peptide-stabilized AuNPs for biomedical applications.

## 1. Introduction

The rational design of nanostructured materials for industrial and biomedical applications revolves around tuning the size and shape of nanoparticles as well as the conscientious selection of passivating ligands on the surface. While size and shape-control of the nanoparticle core govern the optical, electronic, and magnetic properties [1,2,3,4,5,6,7], the capping ligands give rise to biocompatibility, solubility in various solvents, and desired functionalities [8,9,10]. Ligands can also impart negative attributes such as unwanted nanoparticle aggregation and inherent toxicity [11]. For these reasons, ligand selection and strategies used to modify them on nanoparticle surfaces are of paramount importance. The current study reports on a facile strategy to tailor peptide capping agents to improve the solubility, stability, and biocompatibility of gold nanoparticles (AuNPs).

A diverse array of ligands have been employed to stabilize AuNPs such as proteins, DNA, peptides, polymers, and alkanethiols [4,12,13,14,15,16]. The molecular level interactions between these ligands and the AuNP surface occur through non-covalent electrostatic interactions, hydrophobic interactions, and direct covalent attachment [17]. The most extensively studied metal-ligand interactions are between thiols and AuNPs, however, much of this work has focused on nonpolar alkanethiols that are insoluble in water [1,2,6,18,19]. To improve their chemical versatility in aqueous environments, thiols with anionic or cationic hydrophilic groups such as 2-mercaptoethanesulfonate, (dimethylamino)ethanethiol hydrochloride, mercaptoundecanoic acid, or thiolated-poly(ethylene glycol) linkers have been employed [20,21]. Natural thiols such as cysteine (Cys) [12], reduced glutathione (GSH) [13,22,23,24,25,26] and synthetic Cys-peptide analogs have been used for stabilizing nanoparticles [3,25,27,28,29,30,31,32]. The structural and functional features of these peptide-capped AuNPs make them ideal for use as imaging agents [14,33], for integration into bioanalytical sensors [4,34], and as cellular mimics for delivery of chelating agents [13,15,24,35,36] or as cancer drugs [37,38]. Recent work has shown that with the appropriate architectural design, peptide-stabilized AuNPs can also resemble and serve as artificial enzyme mimics or nanoenzymes [39,40,41].

**Scheme 1:**
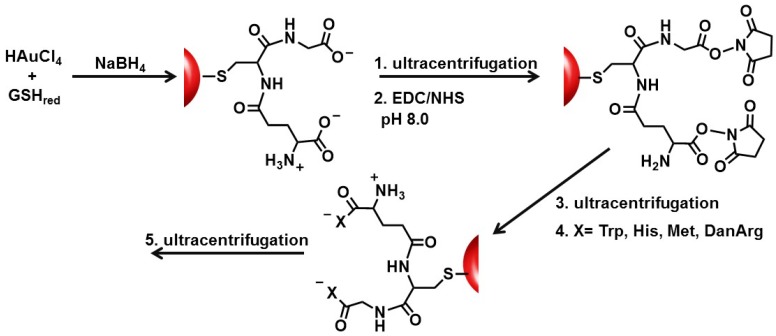
Peptide growth and modification on AuNP supports using GSH as a synthon.

The synthesis of peptide-capped nanoparticles [25,27,28,29,30,32] involves attachment of the peptide through a single terminal thiolate or by multiple Cys within the same peptide framework [3,27,29,30,31,32]. These procedures involve the addition of pre-formed peptide sequences to gold colloids [4,30,34] or to gold salt in the presence of a reducing agent [2,6,14,18,36]. GSH-capped AuNPs were coupled with nonpolar (tryptophan (Trp) and methionine (Met)) and polar basic (histidine (His) and dansyl-labeled arginine (DanArg)) amino acids to produce Au-GSH-(X)_2_ (X=Trp, Met, His, and DanArg) nanoparticles (Scheme 1). This synthetic strategy also involves using ultracentrifugation concentrators for separation of unbound peptides, activation agents, and amino acids after each sequential step in the synthesis. This is important since excess thiols result in the decomposition of AuNPs [39,40] and impurities can contribute to toxic effects upon nanoparticle exposures as well as influence nanoparticle-biological interactions [42]. Some of these impurities can also become active participants in unwanted side reactions that impact the intended composition of the ligand structure. These impurities can affect the nanomaterial stability, solubility, and biocompatibility [43]. Pure nanoparticles allowed us to investigate how peptide structure and length affect the criteria mentioned above for bionanomaterials. This versatile synthetic approach is a useful method for evolving a small library of nanoparticles with tuned peptide sequences for biomedical applications such as optical imaging and drug delivery.

## 2. Results and Discussion

In this study, the basic paradigm exploits the structural features of the GSH synthon for the synthesis of peptide-stabilized AuNPs. GSH possesses a central cysteine thiolate for covalent attachment to the AuNP surface, which orients the terminal polar Glu and Gly residues in an exposed position: (1) for the anionic carboxylic acids to aid in nanoparticle solubility and stability in water; (2) to extend and modify the peptide sequence; and (3) for the growth of two terminal peptide sequences simultaneously (Scheme 1). Though there are 20 natural amino acids to select from, Trp, Met, His, and DanArg were used to modify and lengthen the tripeptide sequence (Scheme 1). Trp and Met were selected to investigate how the incorporation of nonpolar amino acids affects the solubility, stability, and aggregation tendency of the AuNPs. While Met is nonpolar, it has a smaller side chain (S-CH_3_) that is resistant to disulfide formation unlike free cysteine that lead to irreversible nanoparticle aggregation under basic conditions. Conversely, the indole ring of Trp can participate in π-π stacking interactions for nanoparticle self-assembly and is a fluorescent amino acid that could serve as a probe in non-biological environments. The flurophore-labeled DanArg has a polar basic Arg with a naphthalene ring that can also contribute to nanoparticle self-assembly. It is an environmentally sensitive fluorescent probe used in Fӧrester Resonance Energy Transfer (FRET) studies and has a 5-(dimethylamino)naphthalene group that resembles thioflavin, a diagnostic dye used for detecting folded proteins [44,45]. Although DanArg was incorporated for future molecular recognition studies, here DanArg and Trp was utilized as probes to determine coupling efficiency on GSH-capped AuNPs. Conversely, the polar basic His residue is expected to enhance water-solubility even with a slightly bulky imidazole side chain. The incorporation of hydrophobic or positively charged amino acids is expected to shield the gold nanomaterials against nanoparticle-biological interactions for use in biomedical applications.

### 2.1. Synthesis of Au-GSH

**Figure 1:**
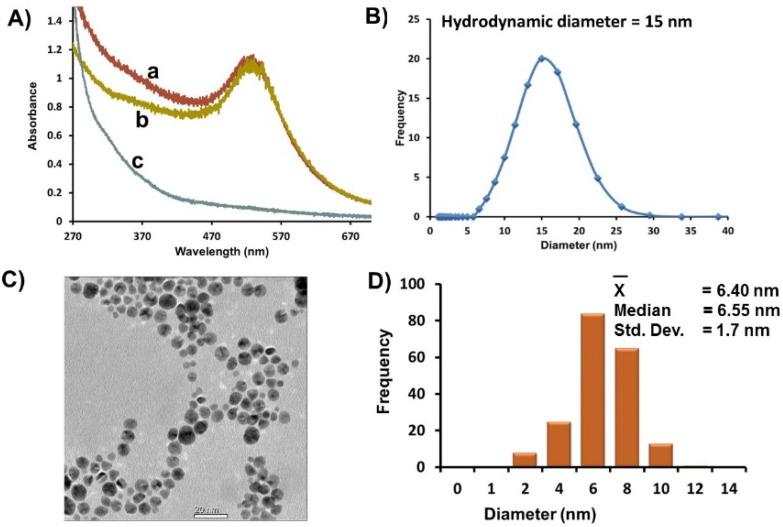
(**A**) Representative UV-Vis spectra of Au-GSH (a) before purification, (b) after purification by ultracentrifugation, and (c) of a brown permeate solution containing smaller nanoparticles removed after ultracentrifugation. (**B**) Average hydrodynamic diameter based on DLS measurements of Au-GSH. (**C**) Representative TEM image. (**D**) Distribution histogram of Au-GSH (Scale bar = 20 nm). All samples were in 10 mM sodium phosphate buffer pH 8.0.

A 2-fold excess of GSH was added to an aqueous solution of HAuCl_4_ that rapidly changed from yellow to a cloudy colorless solution. This color change is consistent with the reduction of Au^III^ to Au^I^ and the formation of Au^I^-SR polymeric species over 30 min [46]. Reduction of the Au^I^-SR intermediate species to Au^0^ occurs upon drop wise addition of a 10-fold excess of NaBH_4_ to yield an initial brown solution that turns maroon-red over the addition period. The UV-Vis spectra of the resulting Au-GSH nanoparticles have a surface plasmon resonance (SPR) band at λ_max_ 521 nm characteristic of spherical nanoparticles (Figure 1A, a). Au-GSH was purified extensively by ultracentrifugation using Vivaspin 20 column concentrators containing a PES membrane of nominal MWCO (10 K) designed to remove 98% of free salts, ligands, and unreacted ions. The UV-Vis spectra of purified Au-GSH remain unchanged with no signs of aggregation (Figure 1A, b). A UV-Vis spectra of the pale brown permeate collected after the first round of concentration and washing showed a weak SPR band as a shoulder that is consistent with smaller AuNPs < 3 nm in diameter (Figure 1A, c) [47]. After several washes, a colorless permeate with no discernible SPR is observed that are characteristic of smaller nanoparticles. Thus, ultracentrifugation is an efficient method for improving the polydispersity of the nanoparticles.

The water-solubility of Au-GSH nanoparticles suggest that GSH is covalently attached to the gold surface through the Cys-thiolate as evidenced by the lack of an -SH stretch at 2540 cm^−1^ in the infrared spectra (Figure S1, A and B) [32,33,43]. An -OH stretch was also not observed since the terminal carboxylic acids of the Glu and Gly resides are deprotonated at pH 8.0 (Figure S1, A and B). In addition, a broad -NH stretch and slight shift to lower stretching frequency was observed in the amide region for Au-GSH (-NH = 3252 cm^−1^) compared to free GSH (-NH = 3266 cm^−1^). The lower -NH stretch falls outside the typical amide A region (~3450–3270 cm^−1^) that is characteristic of an -NH stretch of a very strongly H-bonded amide group [3,27]. When comparing the amide I (C═O symmetric stretching from amide bonds, CN symmetric stretching (ν_s_(CO) and ν_s_(CN)), and NH_3_^+^ antisymmetric bending band δ(NH_3_^+^)) and amide II (-NH in-plane bend and CN symmetric stretching vibrations (δ(NH) and ν_s_(CN)) modes of free GSH and GSH-bound AuNPs, significant shifts to lower stretching and bending frequencies are observed with free GSH (Figure S1, A and B). The amide II shifts by ~55 cm^−1^ while the amide I shifts much less in free GSH. The shift to higher amide I and II stretching frequency confirms GSH binding to the nanoparticle surface. The amide I bonding mode of free GSH is similar to peptide arrangements with β-sheet formations from intermolecular hydrogen bonding interactions. Upon binding to the AuNP surface the intermolecular hydrogen bonding interactions in the β-sheets are disrupted as evidenced by the large shift in the amide II bonding mode [48,49]. That is, there is an increase in the contribution of the -NH bending vibration that leads to higher frequencies. The disruption of β-sheet formations is also observed in the amide I bonding mode of Au-GSH, which is similar to peptides with secondary structures that form unordered arrangements [48,49].

A homogenous distribution of spherical Au-GSH nanoparticles with average diameter of 6.4 ± 1.8 nm was observed in the TEM (Figure 1C,D). While there was a small percentage of overlapping AuNPs, in general, close nanoparticle-nanoparticle (np-np) interactions with distinct interparticle spacing (1.8 nm ± 1.2 nm) were observed (Table S1). This is consistent with a layer of GSH around each nanoparticle and explains the large average hydrodynamic diameter (15 nm) measured by DLS of AuNP solutions (Figure 1B). It is important to note that while close np-np interactions and large hydrodynamic diameters were observed, there were no significant red-shift of the SPR that would suggest nanoparticle aggregation and instability.

### 2.2. Derivatization of Au-GSH Nanoparticles

Au-GSH nanoparticles present an opportunity to further modify the GSH synthon for the preparation of pentapeptide-capped AuNPs. This is similar to solid phase peptide synthesis but on a metal nanoparticle support and allows for simultaneous growth and modification of two peptide sequences. This was achieved by activating the carboxylic acids of purified Au-GSH with a 4-fold excess of EDC/NHS coupling agents (Scheme 1). The resulting NHS-activated Au-GSH nanoparticles were purified by ultracentrifugation in 10 mM sodium phosphate buffer pH 8.0 before a 10-fold excess of Met, His, Trp, or DanArg was added (Scheme 1). This is followed by a final round of purification to remove unconjugated terminal amino acids. The purity of the material was assessed by TLC, UV-Vis, fluorescence, and ^1^H-NMR spectroscopies (*vide infra*).

**Figure 2:**
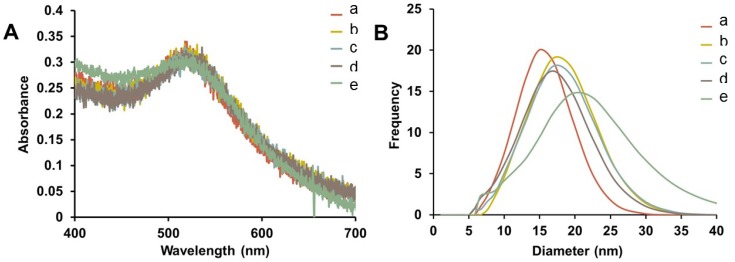
(**A**) Representative UV-Vis spectra and (**B**) hydrodynamic diameter comparison from DLS of AuNPs (a) Au-GSH, (b) Au-GSH-(Trp)_2_, (c) Au-GSH-(Met)_2_, (d) Au-GSH-(His)_2_, and (e) Au-GSH-(DanArg)_2_ in 10 mM sodium phosphate buffer at pH 8.0.

Representative UV-Vis spectra of purified Au-GSH-(X)_2_ (X=Trp, Met, His, and DanArg) nanoparticles show minimal change in the SPR after surface modification indicating that the optical and electronic properties remain unchanged and non-aggregated (Figure 2A). Interestingly, the Au-GSH nanoparticles (15 nm) have the smallest hydrodynamic diameter compared to the pentapeptide derivatives (Table S1) and is consistent with peptide modification and elongation. In the case of Au-GSH-(Trp)_2_ and Au-GSH-(DanArg)_2,_ the hydrodynamic diameter was 19–20 nm (Figure 2B and Table S1). Trp and DanArg non-polar indole and naphthalene substituents allow for π-π stacking interactions between neighboring nanoparticles, hence a large hydrodynamic diameter was observed in solution. Slightly smaller hydrodynamic diameters of 17–18 nm were observed for Au-GSH-(Met)_2_ and Au-GSH-(His)_2_ derivatives (Figure 2B). Au-GSH-(Met)_2_ has nonpolar Met residues with small S-CH_3_ groups that allow for minimal ligand-ligand interactions between adjacent nanoparticles. While Au-GSH-(His)_2_ has an aromatic imidazole ring that could form π-π stacking interactions, its positively charged imidazole ring minimizes charge-charge interactions. Lastly, although peptide sequences with terminal Cys and guanidino groups of Arg are known to cause np-np bridging interactions [4], here no such interactions are observed since DanArg is added after coupling to the GSH-passivated AuNPs. This synthetic methodology is extremely beneficial for preparing tailored peptide-stabilized AuNPs with varying sequences without compromising the structural design of the ligands.

Representative TEM images confirmed the nanoparticle core did not change in size or morphology upon surface modification of Au-GSH (Figure S2). Average diameters were between 6.4 to 7.0 nm depending on the batch of Au-GSH precursor made. Although a TEM of Au-GSH-(DanArg)_2_ was not taken the diameter is expected to be similar. More importantly, the close np-np interactions observed by DLS were apparent in the TEM images. The edge-to-edge interparticle distance measured between adjacent pentapeptide-capped nanoparticles was ~2.0 nm (Table S1). This distance was less than the theoretical estimate of ~3.0 nm, suggesting there is slight interdigitation of the peptide ligands between neighboring nanoparticles. However, interdigitation of peptide ligands due to drying effects is also a possibility.

ATR-FTIR studies also confirmed the presence of the tripeptide and pentapeptide ligand shells and were used to examine the type of ligand interactions involved in nanoparticle stability. The ATR-FTIR spectra of the peptide-stabilized AuNPs showed broad spectral features consistent with high purity nanomaterials with minimal surface ligands (Figure S1). Although weaker, overall the -NH stretches were similar and shifted to higher stretching frequency when compared to GSH (3266 cm^−1^). The amide stretches of Au-GSH-(Trp)_2_ (3288 cm^−1^), Au-GSH-(Met)_2_ (3271 cm^−1^), Au-GSH-(His)_2_ (3301 cm^−1^), and Au-GSH-(DanArg)_2_ (3301 cm^−1^) were observed between amide A and B regions of the infrared spectra and are characteristic of H-bonded -NH groups (Figure S1, C–F). The slight shift to higher stretching frequency suggest that the pentapeptide sequences do not form hydrogen bonding interactions with the nanoparticle surface as seen previously [3,27]. The amide I and II bonding modes of the pentapeptide-capped AuNPs overall range between ~1669–1525 cm^−1^. These bonding modes are higher (≥20 cm^−1^) for pentapeptide-capped AuNPs with polar basic amino acids than those with nonpolar Trp and Met residues and Au-GSH nanoparticles. This higher shift is consistent with peptide sequences with hydrogen bonding interactions in β-turns [48,49]. Sequences with nonpolar residues with lower amide I bonding modes suggest that these peptides on the AuNPs have secondary structures with random arrangements [48,49]. In contrast, the amide II bonding mode is significantly lower for the pentapeptide-capped AuNPs than Au-GSH, suggesting that hydrogen bonding interactions have more significant contributions on the -NH bending vibration. This is consistent with the solubility and stability differences observed between AuNPs with tripeptide and pentapeptide sequences (*vide infra*).

### 2.3. Determining Nanoparticle Purity and Confirming Conjugation

Nanoparticle purity is important when it comes to more precise determination of optical and electronic properties, in assessing structure-function relationships in toxicology studies, and for determining the functional role of the ligand on the nanoparticle surface. A number of techniques can be used to purify nanoparticles such as extensive washing with solvents or fractional crystallization. In cases where the nanomaterials and ligands are water-soluble, dialysis has shown to be effective. However, it produces a large amount of aqueous waste and is time intensive. In column chromatography the nanomaterials can adhere to the column reducing the yield. Diafiltration has been shown to be an effective method for purifying water-soluble nanoparticles with low polydispersity, high purity, minimal waste generation, and improved yield [47]. In addition, ultracentrifugation using PES membrane-supported concentrators with selected nominal MWCO’s minimize membrane blockage and are also 98% effective in separating biomolecules from nanomaterials [50,51]. In this study ultracentrifugation was also employed as a method of purification during the multi-step surface modification of GSH-capped AuNPs. This is important for: (1) detecting if the terminal amino acids are conjugated to the AuNPs; (2) determining the efficiency of coupling to the GSH-capped AuNPs; (3) evaluating the stability; and (4) investigating nanoparticle toxicity.

**Figure 3:**
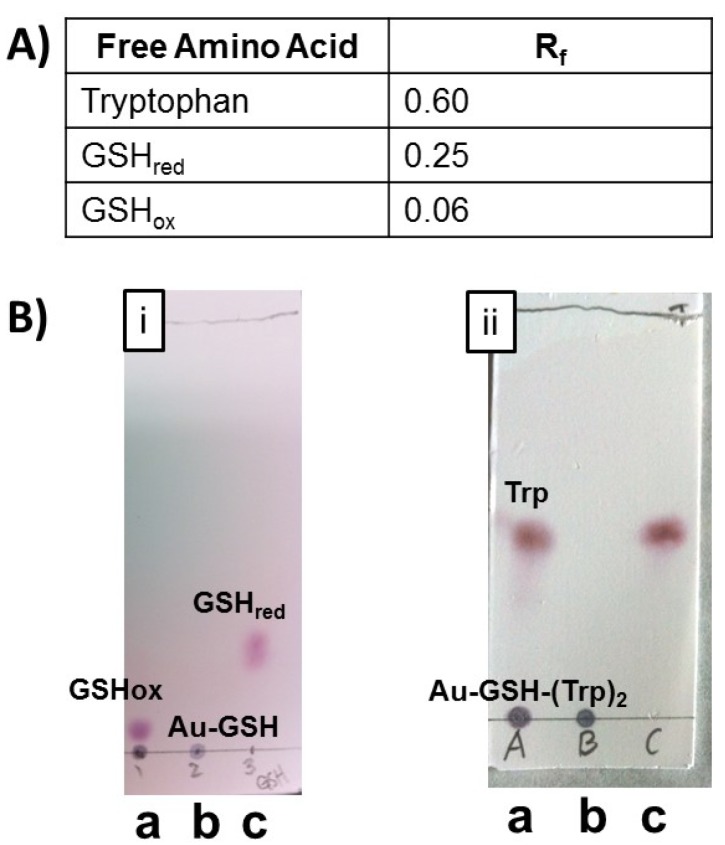
(A) Table with retention factor values for compounds listed on the TLC. (**B**) Representative TLC plates of (i) Au-GSH and (ii) Au-GSH-(Trp)_2_ spotted (a) before purification, (b) after purification by ultracentrifugation, and (c) of free GSH or Trp in butanol/acetic acid/H_2_O (12:3:5) solvent.

To determine if ultracentrifugation concentrators produce high purity peptide-stabilized AuNPs analysis by several spectroscopies were performed. ^1^H-NMR spectroscopy and TLC were used to assess the amount of excess precursor molecules or impurities in the samples. TLC of nanoparticle samples was performed using a mixture of butanol/acetic acid/H_2_O (12:3:5) followed by spraying with ninhydrin. Representative TLC’s of unpurified and purified Au-GSH (Figure 3B, i) and Au-GSH-(Trp)_2_ (Figure 3B, ii) nanoparticles as well as free amino acids and GSH were shown in Figure 3B. Two spots were observed in the TLC with unpurified nanoparticles corresponding to AuNPs, free amino acids or GSH. Whereas, only one spot was observed with purified nanoparticles (Figure 3). This was also observed with all the peptide-capped AuNP derivatives (Figure S3) demonstrating that TLC is a convenient method to confirm nanoparticle purity. The absence of GSH before EDC/NHS coupling chemistry eliminates the possibility of side reactions (Figure 3B, i). UV-Vis and fluorescence spectroscopies were also used to confirmed the absence of Trp, His, Met and DanArg, which absorb in the UV-Vis region (200–330 nm). Analysis was performed on 1 mL concentrated permeate samples collected after ultracentrifugation. No characteristic absorption in this region of free ligands were observed. Analysis of the permeate by fluorescence also showed no spectroscopic evidence of unconjugated Trp (λ_ex_ = 280 nm, λ_em_ = 356 nm) and DanArg (λ_ex_ = 330 nm, λ_em_ = 541 nm) ligands. These techniques confirm that ultracentrifugation produces highly pure materials.

**Figure 4:**
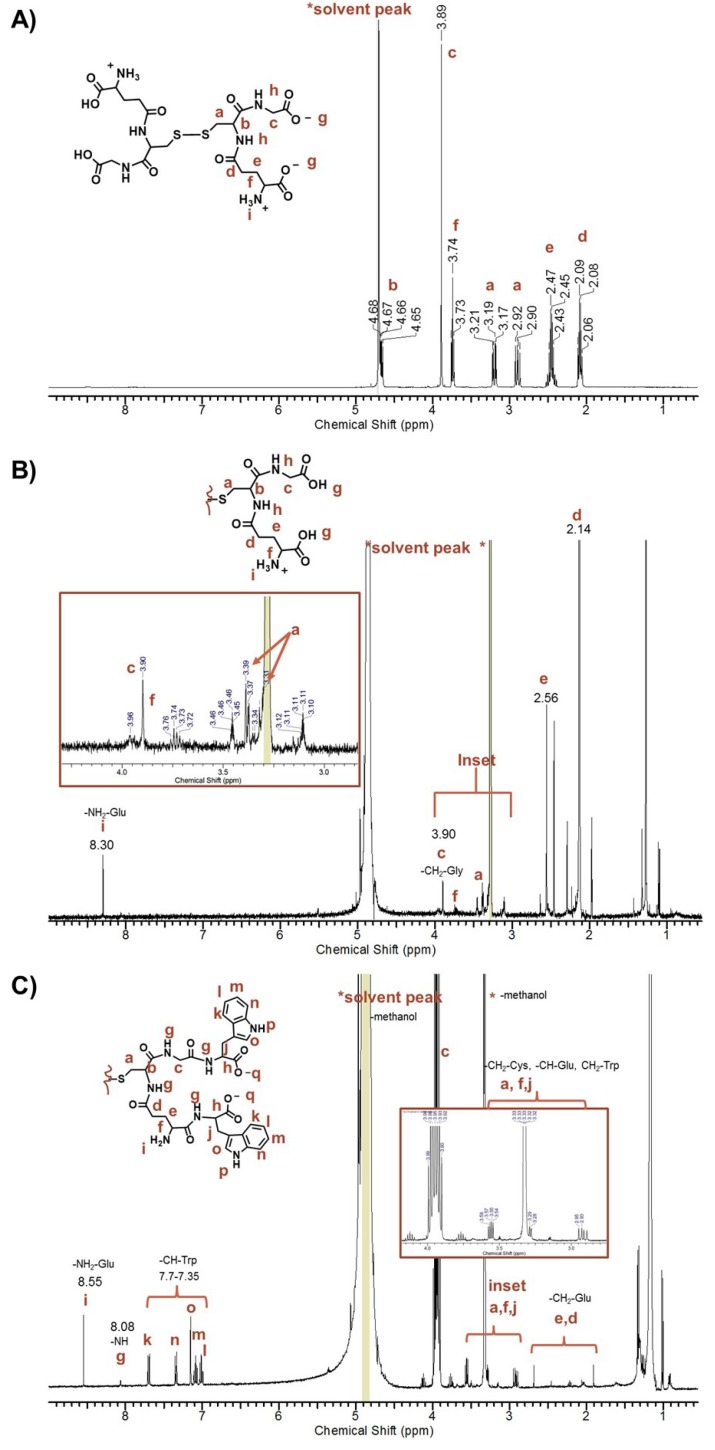
Representative ^1^H-NMR spectra taken in MeOD of (**A**) oxidized GSH, (**B**) Au-GSH etched with concentrated HCl, and (**C**) Au-GSH-Trp_2_ etched with excess cyanide.

The ^1^H-NMR spectra of unpurified peptide-stabilized AuNPs exhibited a complicated spectrum with sharp signals corresponding to protons of the peptide-capped AuNPs and free amino acids (Figure S4, A), which is consistent with free ligands present in the sample [47]. In contrast, the concentrated pure peptide-capped AuNPs in 100% D_2_O showed line broadening and loss of splitting patterns from proton-proton coupling, making it difficult to assign any proton signals. For example, no amide, amine or aromatic proton signals in the 7–8 ppm region of the Au-GSH-(Trp)_2_ derivative were observed (Figure S4, B). Here the free ligand concentration was below the NMR detection limit in the presence of the AuNPs with minimal surface ligands (Figure S4). To confirm conjugation of terminal amino acids, the ^1^H-NMR spectra of samples were taken of AuNPs etched with cyanide in water and then dissolved in methanol (Figure 4 and Figure S5). Cyanide is a well-known etchant that oxidizes Au° to Au^I ^ and was used to cleave the peptide from the AuNP surface [52,53]. These samples showed proton signals between 7–8 ppm corresponding to amines, amides, and aromatic protons. For example, the ^1^H-NMR spectra of etched Au-GSH-(Trp)_2_ in methanol showed characteristic signals of the -CH groups of Trp (7.70–7.35 ppm) shifted downfield from free Trp, amide protons on the peptide backbone (8.03 ppm), and the amine group of Glu residue (8.55 ppm) (Figure 4C). Note that the amide proton signal is small because of its rapid exchange rate [53]. When compared to etched Au-GSH and free oxidized GSH, Au-GSH-(Trp)_2_ showed a set of multiplets between 3.55–2.90 ppm (Figure 4C) that corresponds to the -CH and -CH_2_ groups of Cys, Glu, and Trp on the peptide backbone. The -CH bond (-NH-CH-C(O)-) of Trp was shifted downfield from free Trp and overlaps with the D_2_O signal. While the high signal to noise ratio and the baseline made it difficult to perform integration, these spectral changes in ^1^H-NMR are consistent with amide bond formation upon Trp coupling to the Glu and Gly residues. Although there were small unidentified signals that indicate non-bonded impurity, based on the splitting pattern the predominant product was determined to be the disulfide species, Trp_2_-GS-SG-Trp_2_, which has a white appearance.

With respect to the other pentapeptide-capped AuNP derivatives, upon cyanide etching the resulting disulfide species had limited solubility in methanol. Therefore, these derivatives were etched with concentrated HCl where they have greater solubility for NMR studies in MeOD. The resulting HCl etched product is yellow indicating oxidation of Au^0^ to Au^III^ ions. For example, an HCl etched sample of Au-GSH-(His)_2_ showed -CH (7.54 and 8.94 ppm) signals consistent with that of the imidazole ring and a downfield shift of the -CH triplet signal (-NH-CH-C(O)-) of free His from 3.93 to 4.39 ppm (Figure S5, A and B). The -CH protons of the imidazole was also shifted downfield compared to free His (7.34 ppm and 8.60 ppm) (Figure S5, A and B). Overall when compared to GSH cleaved from the AuNP surface and unconjugated GSH as well as His these spectral changes confirm amide bond formation between the His and the Glu and Gly amino acids (Figure 4 and Figure S5). The relative intensity of these proton signals in the ^1^H-NMR spectra was consistent with the formation of predominantly GSH-(His)_2_ in the presence of Au^III^ ions. That is, there was no evidence of mixed species such as GSH-(His) or GSH-(His)_3_. Evidence that GSH was in a reduced or oxidized state in GSH-(His)_2_ could not be determined by ^1^H-NMR since both species would be soluble under acidic conditions in MeOD. Similarly, coupling of Met and DanArg residues to Au-GSH was also observed in the ^1^H-NMR spectra of Au-GSH-(Met)_2_ and Au-GSH-(DanArg)_2_ derivatives. This was evident by the downfield shift of the -CH proton (-NH-CH-C(O)-) of the Met from 3.90 to 4.16 ppm and splitting of a -CH protons of DanArg linker as a pair of multiplets shifted from 3.83–3.79 ppm to 4.05–3.95 ppm (Figure S5, D and F). In addition, signals corresponding to the protons on the aromatic rings (6–8 ppm) and the N(CH_3_)_3_(2.44 ppm) were also observed. Other minor unidentified signals in the ^1^H-NMR spectra of Au-GSH-(DanArg)_2_ was attributed to decomposition or impurities. Regardless, the spectral changes support amide bond formation of DanArg or Met to the Gly and Glu residues.

**Figure 5:**
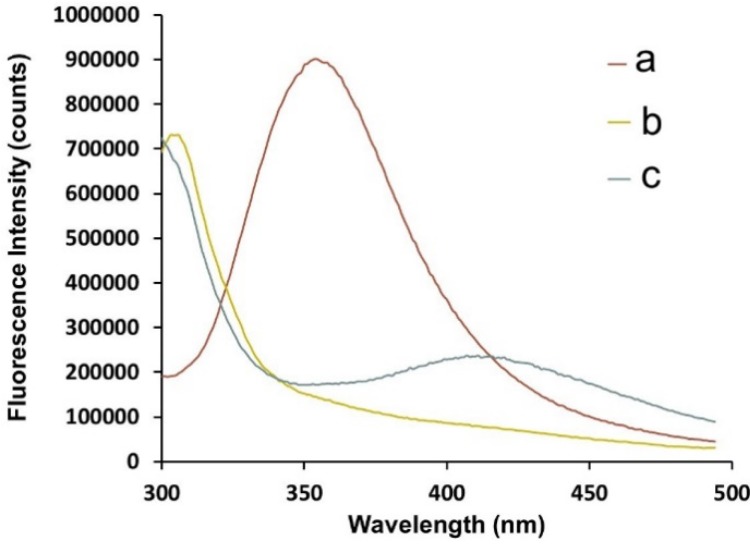
Representative fluorescence spectra of (**a**) unpurified Au-GSH-(Trp)_2_, (**b**) purified Au-GSH-(Trp)_2_ with an O.D. of 0.60 before cyanide etching in 10 mM sodium phosphate buffer pH 8.0 and (**c**) after cyanide etching in methanol.

Fluorescence spectroscopy also confirmed bioconjugation of Trp and DanArg amino acids to the Au-GSH upon decomposition with cyanide. Whereas ample amount of free Trp was seen in unpurified Au-GSH-(Trp)_2_ (Figure 5a), the fluorescence spectra of purified Au-GSH-(Trp)_2_ (Figure 5b) and Au-GSH-(DanArg)_2_ (Figure S6, a) exhibited no discernable emission bands indicative of free Trp or Dan Arg. Purified DanArg and Trp conjugated AuNPs showed quenched fluorescence due to the short donor-acceptor distance between the gold core and fluorophores. This is consistent with other examples where the degree of fluorescence quenching is dependent on nanoparticle size, surface to volume ratio, donor-acceptor distance, and quantum yield of the dye [54,55,56]. However, upon etching Au-GSH-(Trp)_2_ or Au-GSH-(DanArg)_2_ emission bands at 411 nm (Figure 5c) or 416 nm (Figure S6, c) were observed respectively. In addition, a shift in the λ_max_ emission of the fluorophores was observed compared to free ligands. The fluorescence band of GSH-(Trp)_2_ red-shifted (Δ in λ_max_ 46 nm) while it blue-shifted (Δ in λ_max_ 125 nm) for GSH-(DanArg)_2_ from free Trp and DanArg respectively. The red-shifted fluorescence band results from formation of Trp_2_-GS-GS-Trp_2_ upon cyanide etch and oxidation of Au^0^ to Au^I^ ions. The blue shift of GSH-(DanArg)_2_ was hypothesized to result from cyanide reactivity with the arginine residue to form an anion-ionophore. This adduct exhibits a blue-shift and fluorescence enhancement similarly to that observed with dansylated compounds used in cyanide sensing applications [57]. Here, the fluorescence of etched samples of GSH-(DanArg)_2_ was significantly quenched by the Au^III^ ions but still observed because of fluorescent enhancement in the presence of cyanide ions. Cyanide reactivity with the DanArg was also evident by the lack of a UV-Vis absorption band at λ_max_ = 330 nm for DanArg and is consistent with dansyl incompatibility with strong oxidizing agents such as acids and cyanides.

### 2.4. Determining the Number of Binding Sites and % Coupling on GSH

To demonstrate that this synthetic methodology is feasible for extending and tailoring the GSH synthon, it is important to validate the % coupling. Low coupling efficiency can result from side reactions and reduced number of conjugation sites resulting from steric hindrance of tightly packed GSH ligands on the surface. To estimate the % coupling it was necessary to approximate the amount of GSH ligands on the AuNPs. The synthesis of the AuNPs was done with a 1:2 ratio of Au:GSH. To calculate the amount of ligand coverage on the nanoparticle surface of a certain diameter (D), the optical density (O.D.) of the stock solutions is converted to a concentration using Beer’s Law and Equation (1), where *k* and *a* are 3.32 and 10.8, respectively [58]:

ln ε = k ln D+ a
(1)

The number of entities (N) per mL was determined from Equation (2) using Avogadro’s number, N_A_, and the concentration of the nanoparticles, C:

N = N_A_C (2)

A 6.4 nm Au-GSH-(Trp)_2_ nanoparticle has an estimated 800 gold atoms on the surface. However, due to the relative size of the GSH ligand is unlikely that at 1:1 binding ratio of Au:GSH occurs. We estimate that 304 molecules of GSH are on the surface of the 6.4 nm colloid based on a calculated minimal projection area of 42.3 Å^2^ for GSH [59]. Since each GSH has two terminal carboxylic acid groups there are 608 carboxylic acid groups available for coupling amino acids. For example, based on the O.D. of a 1 mL solution of Au-GSH-(Trp)_2_ (O.D. = 0.60) with AuNPs of an average diameter of 6.4 nm there are theoretically ~16 nmoles of Trp molecules conjugated to the GSH-capped AuNPs.

**Figure 6:**
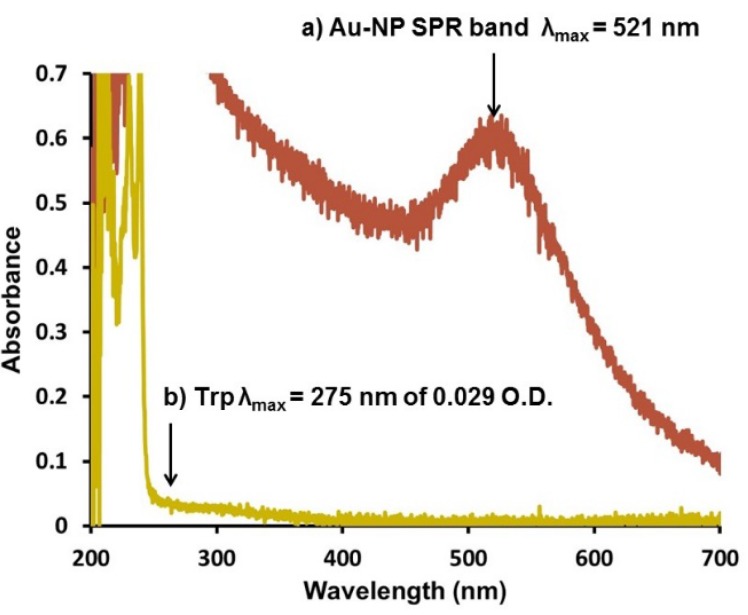
Representative UV-Vis spectra of Au-GSH-(Trp)_2_ nanoparticles (O.D = 0.60, λ_max_ = 521 nm) (**a**) before and (**b**) 20 min after cyanide (15.3 mM) addition in 10 mM sodium phosphate buffer at pH 8.0.

To confirm the amount of Trp actually coupled to the Au-GSH this sample was etched with cyanide (15.3 mM) to decompose the gold core (Figure 6a). Within 20 min of cyanide addition, the AuNP solution becomes colorless and the SPR disappears (Figure 6b). The slow rate of cyanide etch suggests that AuNPs are covalently passivated by the thiolated-peptides. This was followed by the appearance of a new absorption band with an O.D. of 0.029 at λ_max_ 275 nm that is indicative of Trp (Figure 6b). Although the absorption is low, using the O.D. (0.029) and the molar absorption coefficient (5579 M^−1^ cm^−1^) of Trp the number of conjugated Trp was determined to be 5.2 nmol after cyanide decomposition. Based on the theoretical estimate of 16 nmol of Trp on a 6.4 nm gold core a 32.5% coupling efficiency is obtained. That is for each nanoparticle there are 800 gold atoms, 304 GSH, and 198 Trp molecules on the surface. Since an absorption band at 330 nm of DanArg was not observed in the UV-Vis after cyanide etch, a fluorescence matching experiment was performed to determine the coupling efficiency. Using unconjugated DanArg of various concentrations as a reference (Figure S10, Table S2, and Figure S11) the coupling efficiency was determined to be 41.4% for the Au-GSH-(DanArg)_2_ derivative (Supplemental information).

High coupling efficiency for folic acid-GSH-AuNPs has been reported [33], however, these AuNPs were twice as large as the current peptide-capped AuNPs. In contrast, low coupling efficiency (~25%) was observed with 6 nm Au-GSH nanoparticles conjugated with a bulky tin chlorin e6 group [38]. Possible reasons for the estimated low coupling efficiency with the Au-GSH-(Trp)_2_ and Au-GSH-(DanArg)_2_ include: (1) oxidation of the fluorophore-labeled species; (2) fluorescence quenching by oxidation upon cyanide etch; and (3) steric hindrance on the Au-GSH surface. To evaluate these possibilities, the following analysis and control studies were performed. The weak shoulder observed in the UV-Vis at λ_max_ 275 nm upon etching of Au-GSH-(Trp)_2_ suggests Trp oxidation into different compounds with altered spectral properties [60]. Therefore, the molar absorptivity of oxidized Trp would be different and will impact the determination of the coupling efficiency. However, the ^1^H-NMR of the Au-GSH-(Trp)_2_ derivative did not show any proton signals of oxidized Trp eliminating this possibility. In the presence of reducing agents such as NaBH_4_ and GSH, no quenching of the Trp fluorescence or change in the absorption band was observed indicating that Trp was still in a reduced state (Figure S7, B (ii and iii)). Addition of cyanide, oxidized GSH (GSSG), Au^I^-GSH, or Au^III^ ions to unconjugated Trp leads to a significant decrease in fluorescence intensity and change in the absorption of Trp (Figure S7, A (ii and iii) and B (vi and v)) similar to that of oxidized Trp [60]. Insolubility of the oxidized species could also result in low absorption bands. The fluorescence of Trp and DanArg is quenched by the production of gold ions or oxidized GSH from cyanide etch. If the fluorescence is quenched in the presence of these species the coupling efficiency is greater than that estimated by fluorescence and UV-Vis spectroscopies.

^1^H-NMR provided a more accurate assessment of the coupling efficiency where the majority of the etched product was determined to be reduced GSH-(X)_2_ or oxidized X_2_-GS-SG-X_2_. Although there are some minor impurities, proton signals corresponding to free amino acids or a mixture of coupled products (GS-X and GS-(X)_2_) or uncoupled GSH were not observed (Figure 5 and Figure S5). For example, with the Met and His derivatives, only one distinct triplet at 4.16 ppm corresponding to the -CH proton of -HN-CH-C(O) of amino acids was observed. In addition, more than one signal for the -CH_2_ protons on Gly at 3.95 ppm of GSH (which do not overlap with other protons on the ligand framework) were not observed for any of the derivatives. These lines of evidence confirm the absence of free terminal amino acid and other side products (GS-X). That is, the final coupled product is GSH-X_2_ where both the Glu and Gly residues have the same terminal amino acid occurring with 100% coupling efficiency.

### 2.5. The Effect of Peptide Composition on the Stability of Nanoparticles

**Figure 7:**
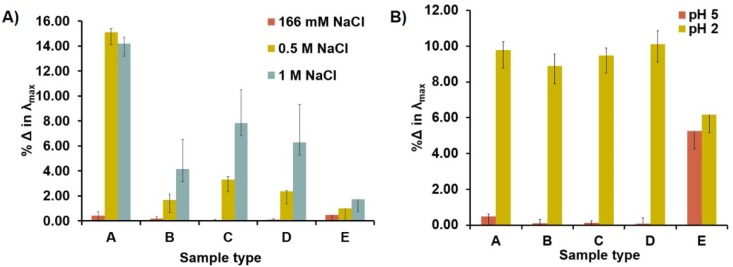
Percent change in λ_max_ of peptide-stabilized AuNPs after (**A**) exposure to 0.17 M, 0.5 M, or 1 M NaCl in 10 mM sodium phosphate buffer pH 8.0 and (**B**) after adjustment of pH to 2 and 5 with 2 M HCl (aq). The sample types are: A) Au-GSH, B) Au-GSH-(His)_2_, C) Au-GSH-(Met)_2_, D) Au-GSH-(Trp)_2_, and E) Au-GSH-(DanArg)_2_. Data mean ± SD reported for n = 3.

Since the ultimate target of these peptide-stabilized nanoparticles are for theranostic applications they must be stable in a range of pH and ionic strengths. Hence, stability studies with the peptide-stabilized AuNPs was performed with AuNPs (O.D. 0.3 or 9.57 nM) exposed to 0.17 M, 0.5 M, and 1 M NaCl for 1 h. The percent change in λ_max_ and O.D. was monitored to assess nanoparticle aggregation and dipole-dipole coupling [61,62]. Under physiological salt concentrations (0.17 M) the percent change in O.D. for all peptide-stabilized AuNPs were 1%–6% and increased significantly at higher salt concentrations (Figure S8, A). Since more distinct differences were seen with the change in λ_max _between the tripeptide and pentapeptide sequences it was used as an indicator of nanoparticle stability. Similarly, using % change in λ_max_, all peptide-capped AuNPs were stable under physiological salt (0.17 M) and basic conditions (Figure 7A). A noticeable 14%–15% red-shift of the λ_max_ was observed for the tripeptide-stabilized Au-GSH nanoparticles in the presence of 0.5–1 M of NaCl compared to the pentapeptide-stabilized Au-GSH-(X)_2_ derivatives with smaller red shifts (% Δ in λ_max_ = 2–8) (Figure 7A). Although the peptide-capped AuNPs were stable under physiological conditions the length and composition of peptide sequence has a significant influence on the stability of the AuNPs. Longer pentapeptide sequences shield the gold core to enhance stability under higher salt concentrations similar to AuNPs with lengthy peptides [4]. Sequences with polar and basic terminal amino acids (Au-GSH-(His)_2_ and Au-GSH-(DanArg)_2_) were more stable than those with nonpolar and hydrophobic residues (Au-GSH-(Met)_2_ and Au-GSH-(Trp)_2_) evident by the small change in λ_max_ (Figure 7A). This demonstrates that hydrophilic residues impart greater stability than hydrophobic residues through hydrogen bonding interactions with the amide protons on the peptide backbone, positive charge on the imidazole nitrogen or ammonium of the guanidino group of Arg, and negatively charged carboxylic acids. This is similar to natural proteins with a hydrophobic core and hydrophilic surface as well as with other tailored peptide sequences coated on AuNPs [4]. The pentapeptide sequences with nonpolar Trp and Met groups also add a layer of protection by shielding the AuNP core through hydrophobic interactions similar to peptide sequences with bulky nonpolar groups such as phenylalanine [4]. Overall based on the % change of the λ_max_ (red-shift of the SPR) nanoparticle aggregation may be ranked in order of decreasing stability as: Au-GSH-(DanArg)_2_ >Au-GSH-(His)_2_ > Au-GSH-(Met)_2_≈ Au-GSH-(Trp)_2_ > Au-GSH.

To evaluate the stability of the AuNPs under varying pH conditions, the acidity was adjusted with 2 M HCl (aq) and the change in λ_max_ was also monitored. All peptide-stabilized nanoparticles were stable at pH 8.0 as is expected since the carboxylic acid groups are deprotonated leaving the peptide-stabilized nanoparticles anionic. In general, reducing the pH to 5 did not have a significant effect on the stability with the exception of Au-GSH-(DanArg)_2_, which had a 6% change in λ_max_ (Figure 7B). Below pH 5 all the nanoparticles aggregate and lose stability as evidenced by the significant increase in the % change in λ_max_ and decrease in O.D. (Figure S8, B). This is expected as the carboxylic acids whose pKa is 4 become protonated [4,63]. Addition of NaOH leads to reversible aggregation and increase in solubility (Figure S9). Upon examining % change in the O.D. it was found that peptide-capped AuNPs with bulky organic substituents had decreased solubility (His, Trp, DanArg) compared to Au-GSH and Au-GSH-(Met)_2_ below pH 5 (Figure S8). Overall AuNPs solubility below pH 5 maybe be ranked in order of decreasing stability as: Au-GSH > Au-GSH-(Met)_2_ > Au-GSH-(His)_2_≈ Au-GSH-(Trp)_2_ > Au-GSH-(DanArg)_2_. Nevertheless, under physiological conditions the nanoparticles were stable and remained soluble, which is important for biological applications and toxicity studies. Although not discussed here, nanoparticle-biological interactions and toxicity studies of Au-GSH-(X)_2_ (X=Trp, His, Met) were performed using a zebrafish model and is the main subject of a separate paper [43]. From these studies, we demonstrated that peptide-stabilized AuNPs purified by ultracentrifugation at each stage of a multi-step synthesis and with an overall positive or neutral charge had low toxicities [43]. Moreover, while the stability of only a few ligands is tested here, these studies suggest that tailoring the peptide-sequence can lead to nanoparticles with greater stability and biocompatibility.

## 3. Experimental Section

### 3.1. General Information

HAuCl_4_·× H_2_O was purchased from Strem Chemicals, Inc., (Newburyport, MA, USA) while reduced l-glutathione (GSH) and α-dansyl-L-arginine hydrochloride were from Sigma-Aldrich Chemical Co., (St. Louis, MO, USA). Sodium borohydride and TLC Silica Gel1B plates were from J. T. Baker and Company (Boston, MA, USA). N-Hydroxysuccinimide 98% (NHS) and histidine were from Acros (Pittsburgh, PA, USA) while 1-(3-Dimethylaminopropyl)-3-ethylcarbodiimide hydrochloride (EDC) and tryptophan were purchased from TCI America (Portland, OR, USA). Methionine was from Alfa Aesar (Ward Hill, MA, USA) and sodium phosphate monobasic monohydroate as well as sodium phosphate dibasic heptahydrate were from BDH Chemicals (Pittsburgh, PA, USA). All chemicals was used as received. Nanopure water was from a Milli-Q ultra-pure system. Ultracentrifugation was performed with a Thermo Scientific Sorvall ST 40R at 4700 rpm using Sartorius Stedim Biotech ultracentrifugal concentrators with a PES membrane (Vivaspin 20, MWCO = 10 K). TLC was performed with Baker-Flex 5 × 20 cm Silica Gel 1B plates with a 200 µm analytical layer using a mixture of butanol/acetic acid/ H_2_O (12:3:5) as the mobile phase.

### 3.2. Syntheses of Au-GSH

HAuCl_4_·× H_2_O (0.025 g, 0.074 mmol) and reduced GSH (0.045 g, 0.15 mmol) were dissolved in H_2_O (10 mL) and stirred vigorously for 30 min. Immediately, the yellow color disappeared and after 30 min a colorless solution appeared. A freshly prepared solution of NaBH_4_ (0.028 g, 0.74 mmol in 5.0 mL of H_2_O) was added one drop/sec under vigorous stirring until the solution changed from a brown to final maroon-red color. The solution was stirred overnight at 25 °C followed by purification by ultracentrifugation at 4,700 rpm using a Vivaspin 20 column and 10 mM sodium phosphate buffer at pH 8.0 (~10 mL × 20) to remove salts and free GSH. The purity of the material was determined by TLC using a butanol/acetic acid/H_2_O (12:3:5) mixture (R_f_ of free GSH_red_= 0.25 and R_f _of GSH_ox_ = 0.06).

### 3.3. Syntheses of Au-GSH-(X)_2_ (X=Trp, His, and Met)

Purified Au-GSH was prepared according to the method described above. To a 15 mL solution of Au-GSH (9.7 × 10^−7^ M) was added EDC (0.021 g, 0.11 mmol) followed by NHS (0.013 g, 0.11 mmol) under vigorous stirring. The reaction was left to stir for 1 h before it was purified by ultracentrifugation with a Vivaspin 20 column at 4,700 rpm with 10 mM sodium phosphate buffer pH 8.0 (~10 mL × 5) to remove excess coupling reagents and side products. The sample was separated into three batches each with 15 mL of NHS-activated Au-GSH nanoparticles (2.1 × 10^−7^ M). To each batch was added 61 µmol of the following terminal amino acids (Trp= 0.012 g, His = 0.013 g, Met = 0.010 g). After 24 h of stirring the sample was purified by ultracentrifugation with copious amounts of 10 mM sodium phosphate buffer at pH 8.0 to remove unconjugated amino acids. The purity of the materials was determined by TLC using a butanol/acetic acid/ H_2_O (12:3:5) mixture (R_f_ of Trp = 0.60, Met = 0.48, His = 0.010).

### 3.4. Syntheses of Au-GSH-(DanArg)_2_

To purified Au-GSH (2.4 × 10^−8^ M) in 10 mM sodium phosphate buffer at pH 8.0 was added a 4-fold excess of NHS (0.0033 g, 0.028 mmol) and EDC (0.0054 g, 0.028 mmol). The reaction was left to stir for 1 h before it was purified by ultracentrifugation at 4,700 rpm with a Vivaspin 20 column with 10 mM sodium phosphate buffer pH 8.0 (~10 mL × 5) to remove excess coupling reagents and side products. A 10-fold excess of DanArg (0.031 g, 0.070 mmol) was then added to the NHS-activated Au-GSH. After 24 h of stirring the sample was purified by ultracentrifugation with copious amounts of 10 mM sodium phosphate buffer at pH 8.0 to remove unconjugated amino acids. The purity of the material was determined by TLC using a butanol/acetic acid/ H_2_O (12:3:5) mixture (R_f_ of DanArg = 0.40).

### 3.5. Physical Measurements

UV-Vis spectra were recorded in water using a USB4000 UV−visible-NIR spectrophotometer (Ocean Optics, Dunedin, FL, USA) with a 1.0 cm path length quartz cell. Infrared spectra was recorded on a Thermo Scientific Nicolet iS10 Smart iTR (Waltham, MA, USA) and fluorescence measurements were performed on a PTI spectrophotometer using Felix32 software. Measurements were taken using a quartz cell at an excitation of wavelength of 280 or 330 nm with a 2 nm bandpass on both monochromators. Dynamic light scattering (DLS) measurements were performed with an LB-550 particle size analyzer (Horiba Co. Ltd., Fukushima, Japan). Transmission electron micrographs (TEM) were acquired on a Tecnai F-20 FEI microscope (Portland, OR, USA). Samples were prepared by drop casting dilute solutions of nanoparticles onto carbon-coated (300 Å) Formvar films on copper grids (Ted Pella, CA, USA). Samples wereair dried overnight before images were collected at an acceleration voltage of 200 kV using a CCD detector. Size and interparticle spacing analysis were performed using ImageJ Software. ^1^H-NMR was performed using a Bruker Advance II 400 mHz instrument (The Woodlands, TX, USA).

### 3.6. Stability and Cyanide Etch Studies

To 1 mL solutions of Au-GSH-(X)_2_ (X=Trp, His, Met, and DanArg) with an O.D. of 0.3 or 9.6 nM was incubated with NaCl (s) to yield final concentrations of 0.17 M, 0.50 M, and 1.0 M NaCl respectively. For the pH study the pH was adjusted to 2 and 5 with 2 M HCl (aq). All samples were incubated for 1 h before the UV-Vis spectra was taken and the percent change in λ_max_ as well as (optical density) O.D. were monitored to assess nanoparticle stability. Cyanide etch studies were performed by incubating 100 µL of AuNPs with 50 µL of 307 mM of KCN that was diluted with 850 µL of 10 mM sodium phosphate buffer pH 8.0 or ethanol after etching to yield final concentrations of 9.6 nM AuNPs and 15.3 mM of KCN.

## 4. Conclusions

In summary, a simple process for preparing water-soluble and physiologically stable peptide-capped AuNPs of high purity is developed. Here a natural tripeptide found in high concentrations in cells is employed as a synthon to tailor and lengthen the peptide sequence with four different terminal amino acids directly on a metal nanoparticle support. This method is unique in that it allows for the simultaneous growth and modification of two peptide chains from a central cysteine residue. This methodology is advantageous as it reduces peptide induced nanoparticle aggregation and instability. Particularly with preformed peptide sequences whose ligand composition have more than one donor group that possess a strong affinity for gold. For example, peptide sequences with two thiolate Cys at different positions or a thiolate and a guanidine group of Arg [3,4,27] are shown to induce nanoparticle-nanoparticle coupling.

TLC, fluorescence, UV-Vis, and ^1^H-NMR spectroscopies confirmed that the high purity nanoparticles are produced with ultracentrifugation using concentrator tubes with a PES membrane that has MWCO of 10 K. This method allows for easy and rapid purification of the peptide-stabilized AuNPs with minimal waste and improved polydispersity. Confirmation of nanoparticle purity is important to determine coupling efficiency as well as for evaluating the effect of peptide length and sequence on stability and nanoparticle-biological interactions.

These peptide-stabilized AuNPs are stable and soluble for weeks at pH 8.0. Stability studies show that these nanoparticles are mostly stable between pH 5–8 and up to 0.17 M of NaCl. Reversible nanoparticle aggregation at pH 2 is proposed to result from protonation of the terminal carboxylic acid groups. Under high NaCl concentrations (>0.5 M) the Au-GSH nanoparticles with the shorter peptide sequence and Au-GSH-(Trp)_2_ and Au-GSH-(Met)_2_ with non-polar side chains show the greatest instability. In contrast, the incorporation of hydrophilic amino acids (His or DanArg) residues aid in protecting the nanoparticle against aggregation. This studies demonstrate that peptide sequence length, structure, and overall charge are important for controlling dipole-dipole interactions.

Using the synthetic and purification approaches discussed here libraries of peptide-templated AuNPs can be developed. This approach allows for lengthening and tailoring the peptide sequence with other amino acids or biomolecules with 100% coupling efficiency. In essence, this design strategy allows one to a develop nanoparticle system that can serve as a protein mimic or have stealth-like behavior to go past the immune system for drug delivery. The nanoparticle core represents the hydrophobic core of amino acids in natural proteins with surrounding hydrophilic residues allowing for water-solubility and non-aggregation. These types of nanomaterials may also have biosensing applications that can integrate an artificial nanoscale structure for protein recognition.

## Supplementary Materials

Supplementary materials can be accessed at: http://www.mdpi.com/1420-3049/19/5/6754/s1.
