# Supplementary material for: A Facile Route to Tailoring Peptide-Stabilized Gold Nanoparticles Using Glutathione as a Synthon
Source: Molecules. 2014 May 23;19(5):6754–75. doi: 10.3390/molecules19056754 (PMC6271629; doi:10.3390/molecules19056754)

Supplementary Materials

Table S1. Comparison of average diameters by TEM and hydrodynamic diameters by DLS of peptide-stabilized nanoparticles.

Sample name	Average diameter (nm) by TEM	Interparticle spacing (nm) by TEM	Average hydrodynamic diameter (nm) by DLS
Au-GSH	6.40 ± 1.75	1.80 ± 1.75	15
Au-GSH-$(Trp)_2$	6.88 ±1.76	1.99 ± 1.72	19
Au-GSH-$(Met)_2$	6.88 ± 1.98	2.16 ± 1.34	18
Au-GSH-$(His)_2$	6.54 ± 1.78	2.02 ± 1.46	17

Figure S1. FTIR spectral overlay of (**a**) reduced glutathione (GSH) ligand, (**b**) Au-GSH, (**c**) Au-GSH-$(Trp)_2$, (**d**) Au-GSH-$(Met)_{2,}$ (**e**) Au-GSH-$(His)_2$, (**f**) Au-GSH-$(DanArg)_2$.

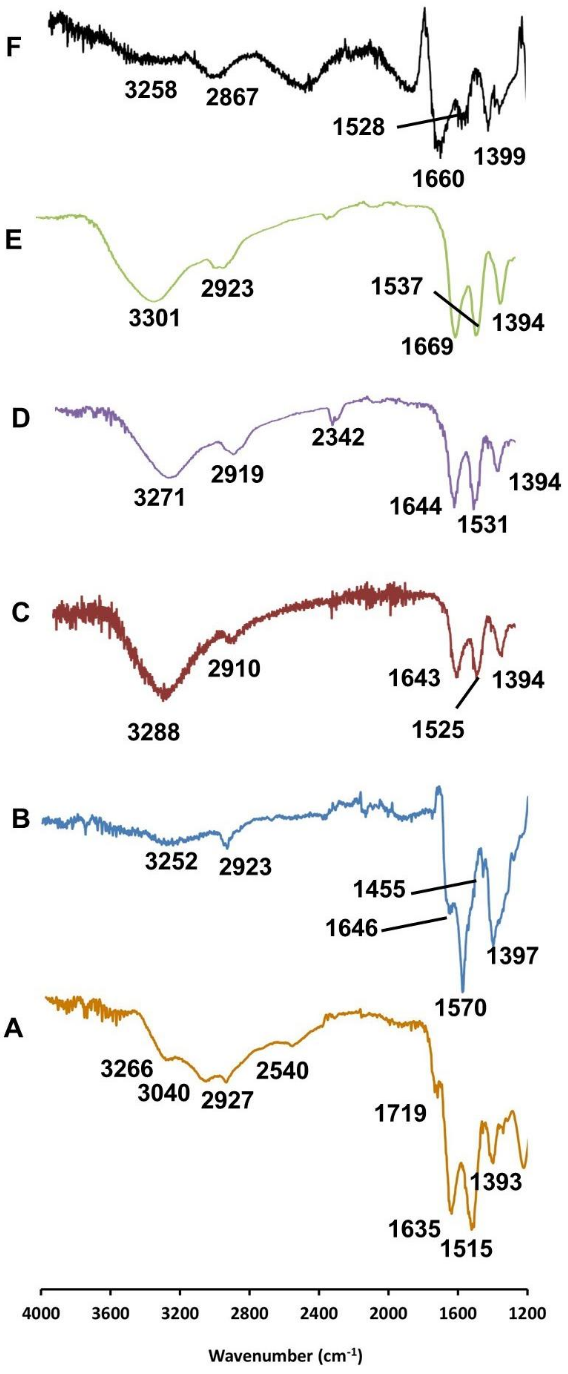

Figure S2. Representative I) TEM micrographs, II) size distribution histograms, and III) interparticle spacing distribution histograms for (**A**) Au-GSH, (**B**) Au-GSH-$(Trp)_2$, (**C**) Au-GSH-$(Met)_2$, and (**D**) Au-GSH-$(His)_2$.

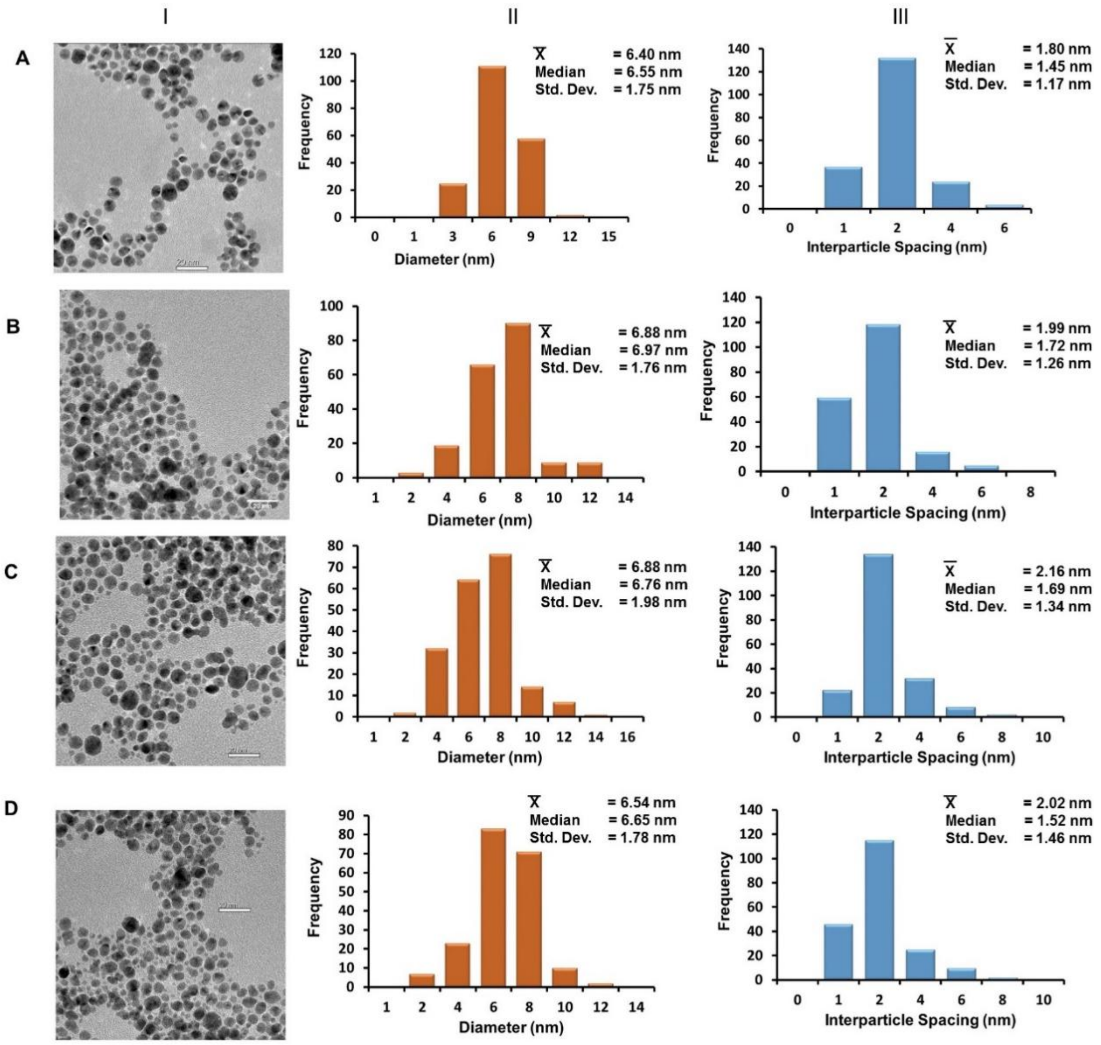

Figure S3. (**A**) Table with retention factors values for compounds listed on the TLC. (**B**) TLC plate of (i) Au-GSH-$(Met)_2$, (ii) Au-GSH-$(His)_2$, and (iii) Au-GSH-$(DanArg)_2$ respectively, (a) before purification, (b) after purification by ultracentrifugation, and c) free Trp, His, Met and DanArg ligands in butanol/acetic acid/H_2O (12: 3:5) solvent.

A

Free Amino Acid	R_f
Methionine	0.48
Histidine	0.01
DanArg	0.40

B

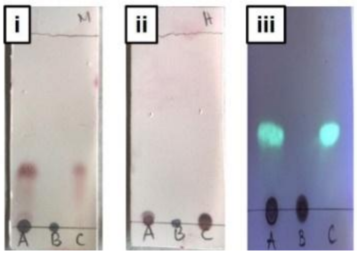

Figure S4. Representative ^{1}H NMR spectra of (**A**) unpurified Au-GSH-$(Trp)_2$ and purified (**B**) Au-GSH-$(Trp)_2$, (**C**) Au-GSH-$(Met)_2$, (**D**) Au-GSH-$(His)_2$, (**E**) Au-GSH-$(DanArg)_2$ in D_2O, (**F**) Au-GSH.

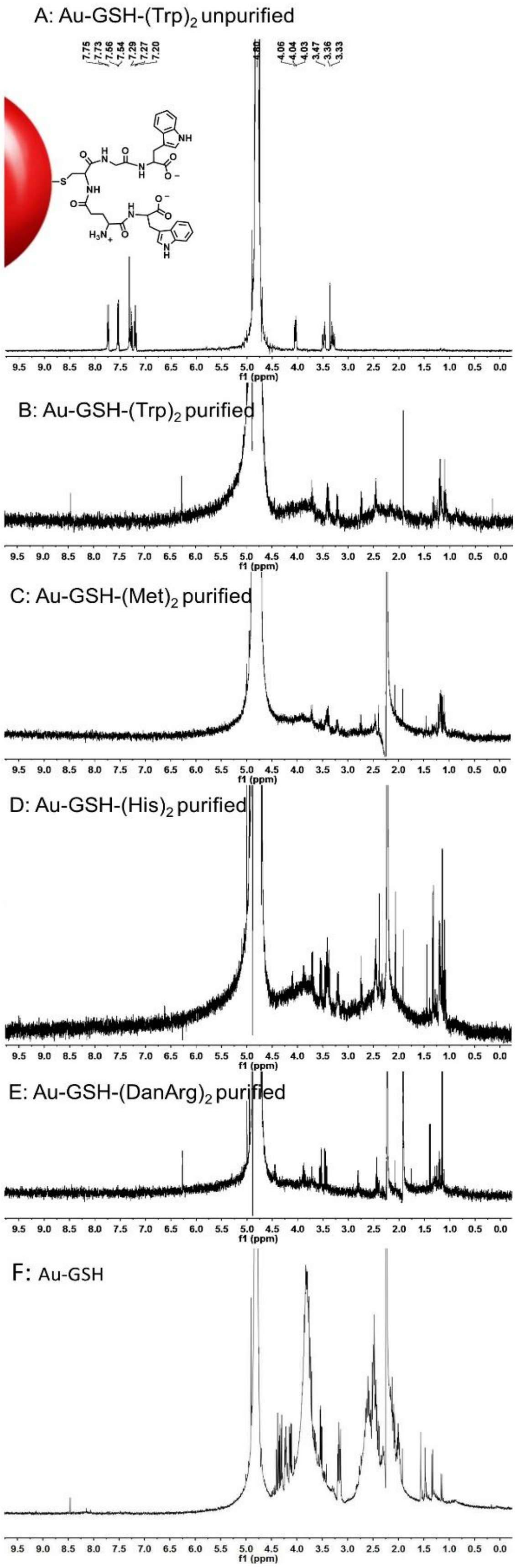

Figure S5. Representative ^{1}H NMR spectra of (**A**) free His, (**B**) Au-GSH-(His)$_2$, (**C**) Met, (**D**) Au-GSH-(Met)$_2$, (**E**) free DanArg, and (**F**) Au-GSH-(DanArg)$_2$ upon etching with concentrated HCl in MeOD.

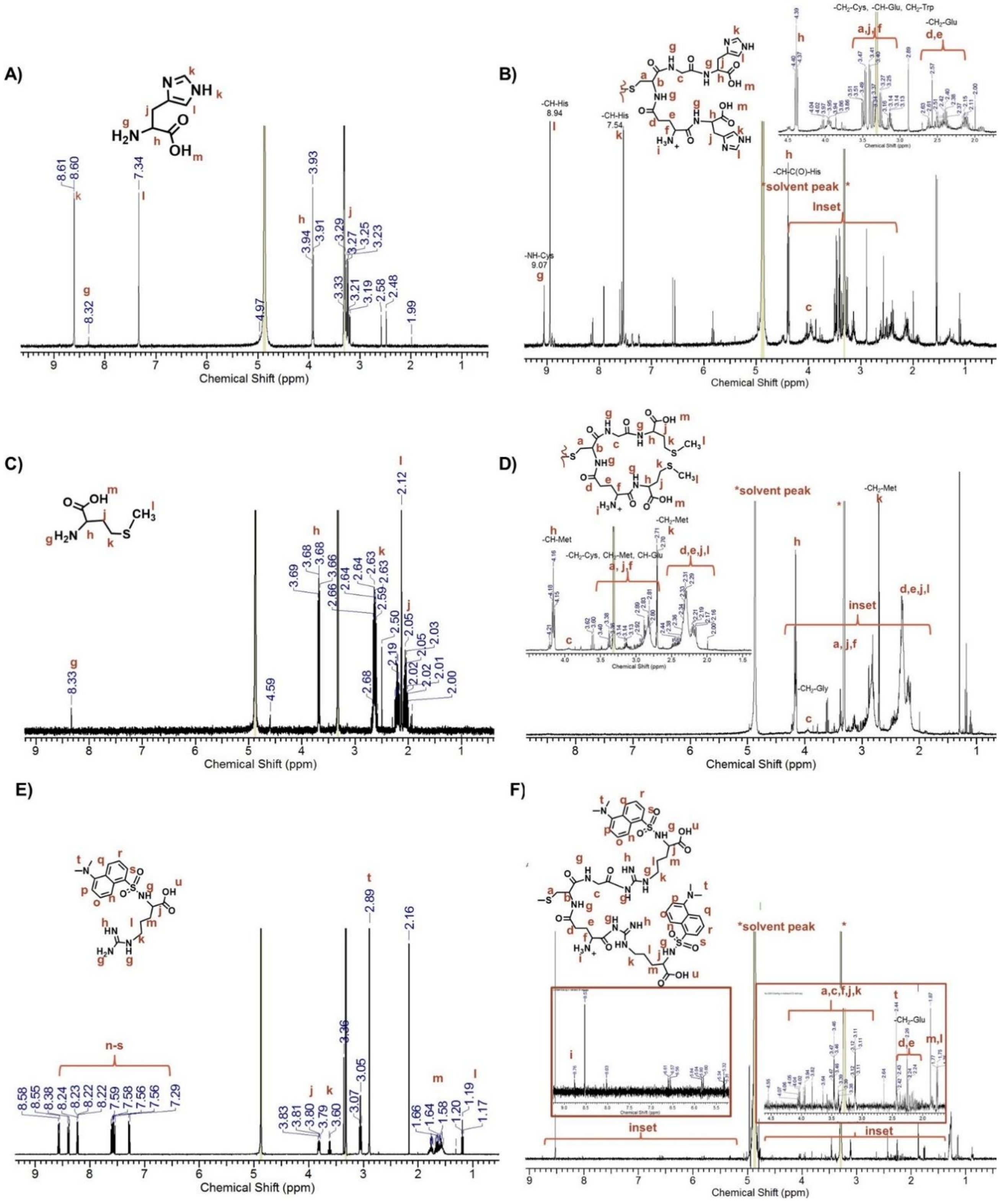

Figure S6. Representative fluorescence spectra of purified Au-GSH-$(DanArg)_2$ (O.D. = 0.3) upon excitation at λ_{max} = 330 nm (a) in 10 mM sodium phosphate buffer pH 8.0 (b) in ethanol, (c) after addition of 15.3 mM cyanide in 10 mM phosphate buffer pH 8.0, (d) after addition of 15.3 mM cyanide in ethanol, and (e) unconjugated 6.3 nM DanArg in 10 mM phosphate buffer pH 8.0.

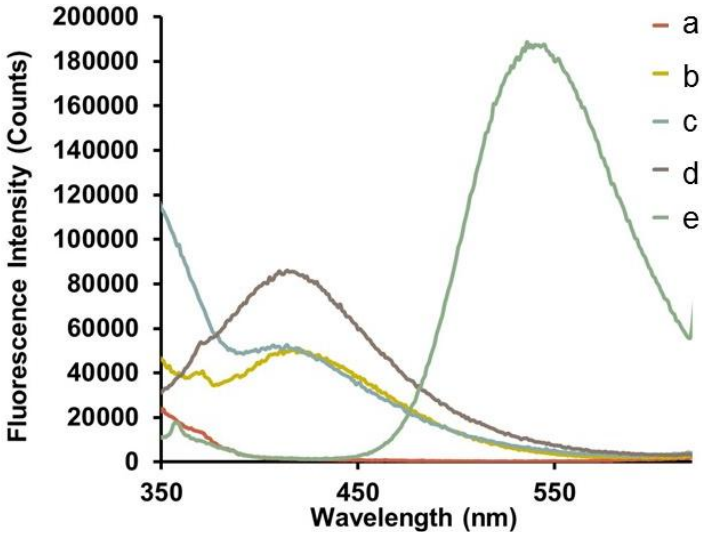

Figure S7. Representative fluorescence spectra of (**A**) i) free Trp, ii) Trp in the presence of 0.23 µM of Au^{3+} ions, and iii) Trp in the presence of 0.23 µM of Au^{3+} ions and 15.3 mM of KCN in 10 mM phosphate buffer pH 8.0. (**B**) i) free Trp, ii) Trp in the presence of 37 µM $NaBH_4$, iii) Trp in the presence of 19 µM GSH_{red}, iv) Trp in the presence of 9 µM GSSG, v) Trp in the presence of Au^1-GSH in 10 mM phosphate buffer pH 8.0.

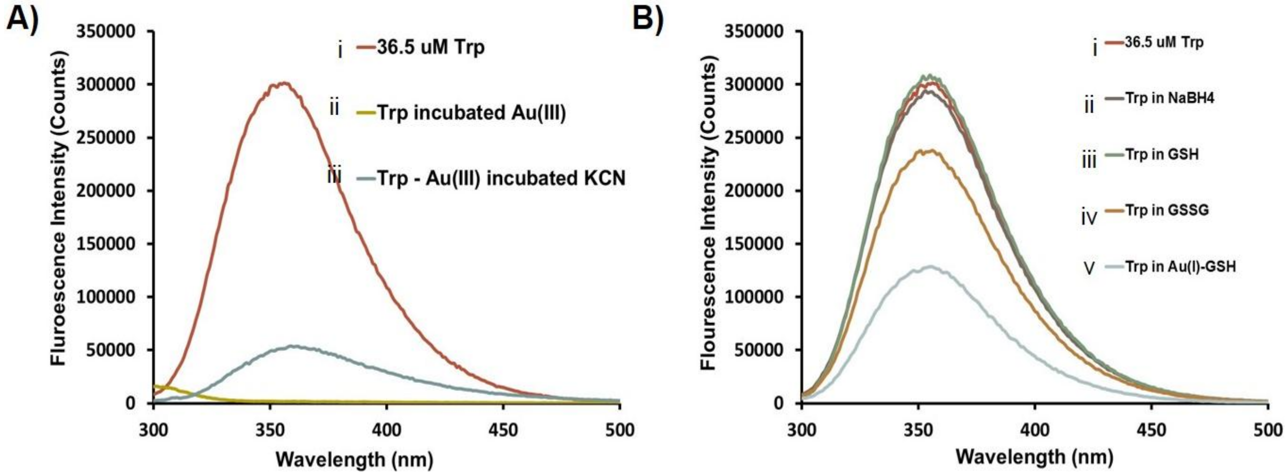

Figure S8. Percent change in O.D. of peptide-stabilized AuNPs after (**A**) exposure to 166 mM, 0.5 M, or 1 M NaCl in 10 mM PBS buffer pH 8.0 and (**B**) after adjustment of pH to 2 and 5 with 2 M HCl (aq). The sample types are: A) Au-GSH, B) Au-GSH-$(His)_2$, C) Au-GSH-$(Met)_2$, D) Au-GSH-$(Trp)_2$, and E) Au-GSH-$(DanArg)_2$. Data mean ± SD reported for n = 3.

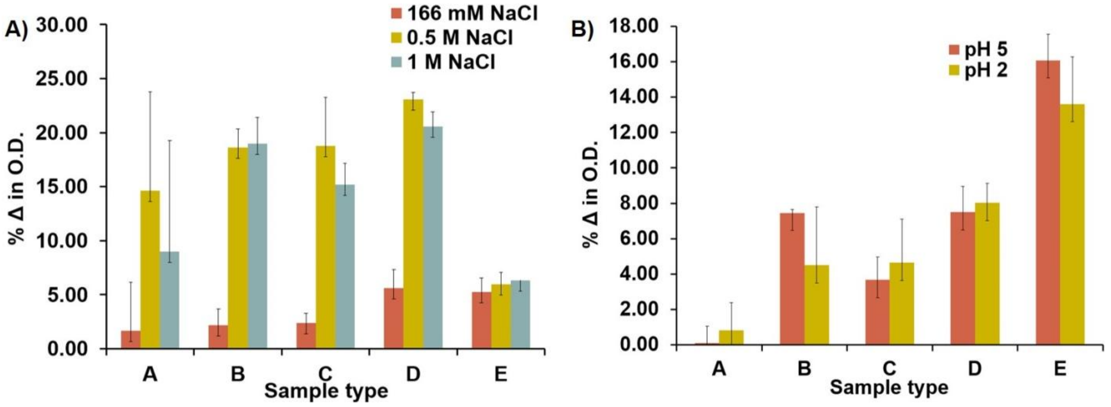

Figure S9. Representative UV-vis spectra of Au-GSH-$(His)_2$ showing reversibility of aggregation up pH adjustment i) sample in initial pH 8.0 ii) pH 2 adjusted with 2 M HCl, iii) addition of 5 M NaOH.

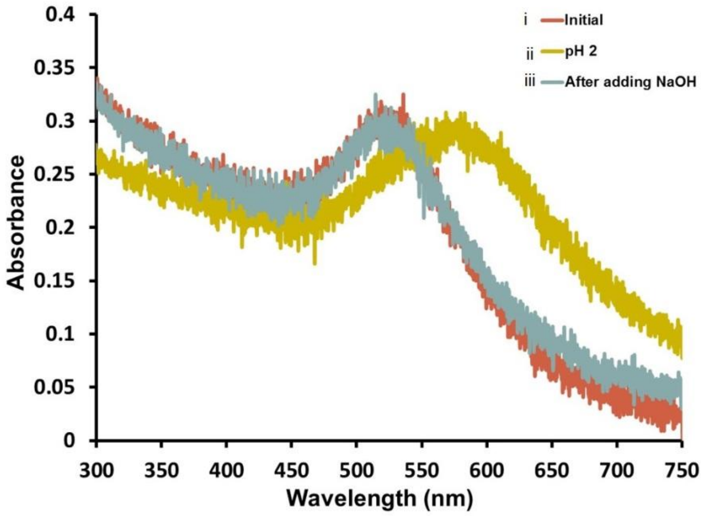

Fluorescence Matching Experiment

To simulate and investigate the effect of gold ions on the fluorescence intensity of etched Au-GSH-$(DanArg)_2$, a fluorescence matching experiment with DanArg and gold ions was performed. We estimate that 304 molecules of GSH are on the surface of the 6.4 nm colloid based on a calculated minimal projection area of 42.3 $Å^2$ for GSH as described in the main text [59]. Using an O.D. of 0.51 of a 100 µL sample of purified Au-GSH-$(DanArg)_2$ a total of 13.3 nmols of surface conjugated DanArg was estimated. From a stock solution of DanArg prepared in in EtOH with a concentration of 31.54 nmol per mL, 1000, 800, 600, 400, and 200 µL were extracted and diluted with EtOH to a final volume of 1000 µL. All samples had concentrations equivalent to 100, 80, 60, 40, and 20% coupling efficiencies, respectively. Fluorescence spectra was recorded and is shown in Figure S10 A and Table S2. To evaluate the effect of the Au^{III} ions on the fluorescence intensity measurements, with

DanArg samples were incubated with 40 µL of 0.005 M M $HAuCl_4$ and fluorescence spectra taken (Figure S10 B and Table S2).

Using this data a correlation between the mole of DanArg (x, nmol) and FLI (y, counts) is expressed by a linear relationship in the equation: $y = 15640.26x + 12289.00$ (Figure S11). Using the FLI of etch $(Au\text{-}GSH\text{-}DanArg)_2$ at 98521 counts at $\lambda_{max} = 410$ nm (Figure S10 B) the experimental amount of DanArg in 100 µL of Au-GSH-$(DanArg)_2$ was calculated using this equation was estimated to be 5.51 nmol. As a result, the calculated coupling efficiency is estimated to be 41.4%.

Figure S10. Different number of moles of DanArg corresponding to 100, 80, 60, 40 and 20% coupling efficiency (**A**) without Au^{III} ions and (**B**) in the presence of Au^{III} ions in EtOH.

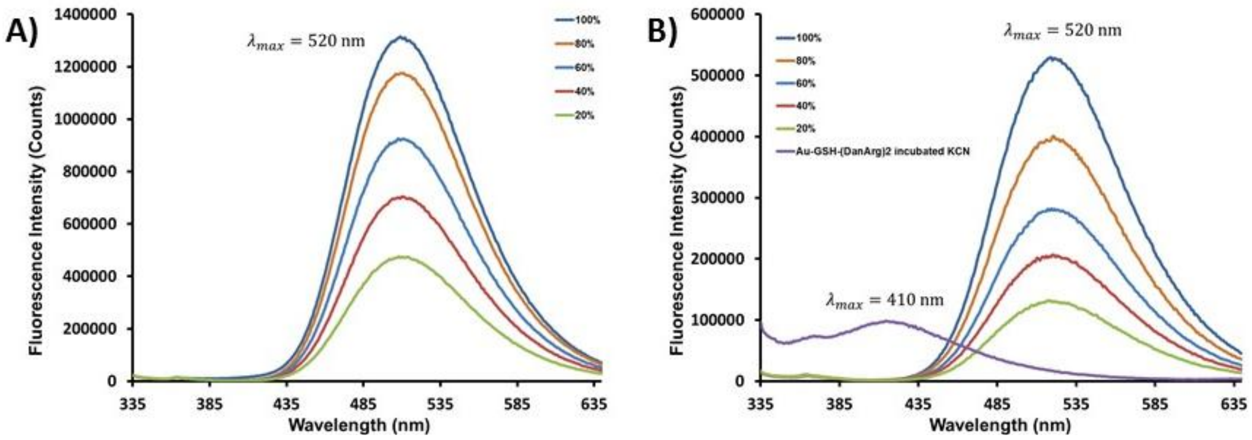

Table S2. Fluorescence intensities of DanArg in the absence and presence of Au^{III} ions.

Stock DanArg/EtOH in 1000 µL sample (µL)	Corresponding % coupling	Mole of DanArg in 1000 µL sample (nmol)	FLI without Au^{III} (counts)	FLI in presence of Au^{III} (counts)
1000	100	31.54	1,272,680	529,719
800	80	25.23	1,147,540	394,048
600	60	18.92	894,816	282,093
400	40	12.61	688,111	203,420
200	20	6.31	461,322	131,816

Figure S11. Correlation between fluorescence intensity and mole of DanArg in the presence of Au^{+3} ions.

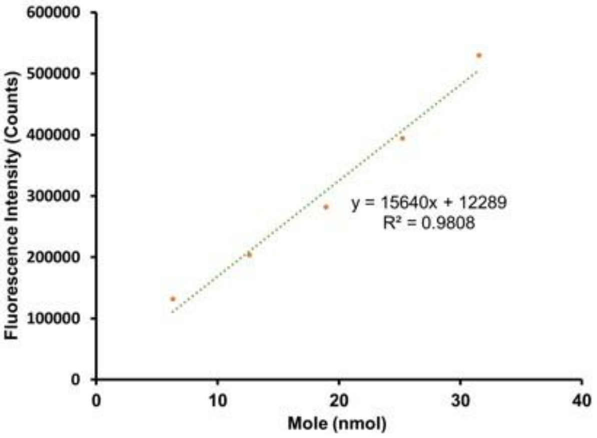

Supplement: Supplementary file 1 [file molecules-19-06754-s001.pdf]
